# Maternal and neonatal outcomes among pregnant women with different polycystic ovary syndrome phenotypes: A cross-sectional study

**DOI:** 10.18502/ijrm.v13i5.7154

**Published:** 2020-05-31

**Authors:** Akramsadat Dehghani Firoozabadi, Razieh Dehghani Firouzabadi, Maryam Eftekhar, Afsar Sadat Tabatabaei Bafghi, Farimah Shamsi

**Affiliations:** ^1^Shahid Sadougui Hospital, Shahid Sadougui University of Medical Sciences, and Health Services, Yazd, Iran.; ^2^Research and Clinical Centre for Infertility, Yazd Reproductive Sciences Institute, Shahid Sadougui University of Medical Sciences and Health Services, Yazd, Iran.; ^3^Abortion Research Center, Yazd Reproductive Sciences Institute, Shahid Sadoughi University of Medical Sciences, Yazd, Iran.

**Keywords:** Pregnancy outcome, Polycystic ovary syndrome, Phenotype, Pregnancy.

## Abstract

**Background:**

Pregnancy is a process associated with various metabolic and hormonal changes, and polycystic ovary syndrome (PCOS) can affect this process.

**Objective:**

This study aimed to evaluate and compare the maternal and neonatal outcomes among pregnant women with different polycystic ovary syndrome phenotypes.

**Materials and Methods:**

In this cross-sectional study, 200 pregnant women with PCOS according to the 2003 ESHRE/ASRM criteria were categorized into four phenotype groups (A-D). The maternal outcomes include gestational diabetes mellitus, pregnancy-induced hypertension, premature rupture of membranes, preterm labor, small-for-gestational age birth, intrauterine growth restriction, intrauterine mortality, preeclampsia, abortion, amniotic fluid disorders, delivery method, and cause of cesarean section were studied between groups. Additionally, neonatal outcomes such as neonatal weight, neonatal recovery, 5-min Apgar score, neonatal icter, the need for NICU admission, the cause of hospitalization, and infant mortality rate were investigated and compared among the groups.

**Results:**

According to the results, phenotype D (37%) was the most common phenotype among the participants. The risk of gestational diabetes was more common in phenotype A than in the other phenotypes, whereas pregnancy-induced hypertension was most common in phenotype B. No significant differences were observed in the neonatal complications among the PCOS phenotypes.

**Conclusion:**

Considering the higher risk of gestational diabetes mellitus and pregnancy-induced hypertension in PCOS phenotypes A and B, women with these phenotypes need more precise prenatal care.

## 1. Introduction

Pregnancy is a process associated with various metabolic and hormonal changes, and the onset of polycystic ovary syndrome (PCOS) can affect this process (1). According to the World Health Organization (2012), women with PCOS are at risk of prenatal complications during pregnancy as well as most pregnancy pathologies, including gestational diabetes mellitus, glucose tolerance disorders, pregnancy-induced hypertension, preeclampsia, fetal growth restriction, preterm delivery, and small for gestational age (2). Previous studies have also reported an increased risk of morbidity and hospitalization in the neonatal intensive care unit in pregnant women with PCOS (3, 4). Some reports have suggested an increase in the incidence of spontaneous abortion and preterm delivery for these women compared with healthy pregnant women (1, 5-8). Due to the importance of pregnancy in these women, especially those with infertility, as well as their phenotypic differences and the limited information available on their pregnancy outcomes, this study was designed to evaluate and compare the maternal and neonatal outcomes among pregnant women with different PCOS phenotypes.

## 2. Materials and Methods

This cross-sectional study was performed on 200 pregnant women under 40 yr old with a history of PCOS according to the ESHRE/ASRM criteria (Rotterdam) (9) who were referred to the obstetrics and gynecology department of Shahid Sadoughi Hospital, Yazd, Iran, between 2017 and 2018.

The exclusion criteria included uterine abnormalities, other causes of hyperandrogenism such as congenital adrenal hyperplasia; malignant ovarian tumors; androgen-secreting tumors; Cushing disease; hyperprolactinemia; chronic diseases such as lupus, kidney, and liver disease; diabetes mellitus; and cigarette, drug, and alcohol use.

Based on the Rotterdam criteria (10), the participants were categorized into four PCOS phenotype groups from the beginning of the prenatal period to delivery:

Type A: Having symptoms of hyperandrogenism, chronic ovulation, and polycystic ovaries

Type B: Having symptoms of hyperandrogenism and chronic anovulation

Type C: Having symptoms of hyperandrogenism and polycystic ovaries

Type D: Having symptoms of chronic ovulation and polycystic ovaries

The participants 'basic characteristics, e.g., age, gravidity, parity, history of abortion and infertility, occupation, history of surgery, use of assisted reproductive techniques, and gestational age, also their maternal outcomes, e.g., gestational diabetes mellitus and hypertension, premature rupture of membranes, preterm labor, small for gestational age, intrauterine growth restriction, intrauterine fetal death, preeclampsia, abortion, amniotic fluid disorders, delivery method, and cause of cesarean section, were recorded. Neonatal information, including weight, neonatal recovery, 5-min Apgar score, neonatal icter, the need for admission, the cause of hospitalization, and the infant mortality rate were recorded in a neonatal complications questionnaire.

### Ethical consideration

The Ethics Committee of the Shahid Sadoughi University of Medical Sciences, Yazd, Iran, approved the proposal of this research (Code: IR.SSU.MEDICINE.REC.1397.114). Written informed consent was obtained from all participants.

### Statistical analysis

Considering a 95% confidence interval, a power of 80%, the frequency of phenotype B (16%) in the participants as the lowest frequency, and a 10% error, a sample size of 200 was determined using the following formula:

$$N=\frac{{\left(Z_{1-\frac{\alpha }{2}}+Z_{1-\beta }\right)}^{2}\times \left[P\left(1-P\right)\right]}{d^{2}}$$

All data were analyzed using the Statistical Package for the Social Sciences (SPSS) software (SPSS Inc., Chicago, Illinois, U.S.A), version 17.0. For the quantitative and qualitative variables, the mean ± standard deviation and the frequency and rate, respectively, were measured and investigated via the Pearson correlation coefficient and Spearman's correlation coefficient tests. A p-value <0.05 was considered significant.

## 3. Results

In this study, 200 pregnant women with PCOS were studied into four phenotype groups (A-D). Two participants from group D were excluded due to unintended pregnancy and the use of abortion induction. Thus, the final study population consisted of 198 pregnant women with a mean age of 29.2 ± 7.7 year. Group D with 37% and group B with 14% had the highest and lowest frequencies, respectively (Table I).

The participants' demographic characteristics are presented in Table II. There was no difference in maternal age (p = 0.32) among the four groups. In group A, the history of abortion (48.9%) and infertility (51.1%) was higher than in the other groups; this difference was significant for the infertility history (p = 0.007) (Table II). The miscarriage rate did not differ statistically in study groups. 37 pregnancies resulted in miscarriage. Maternal outcomes was examined only in cases of ongoing pregnancy.

Gestational diabetes mellitus (GDM) was evaluated via two methods: fasting blood sugar (FBS) measurement and an oral glucose tolerance test (OGTT). Our results showed that the rate of GDM was significantly different among the four groups. In addition, group A had a higher abnormal OGTT than the other groups. The highest GDM was associated with phenotype A and the lowest with phenotype D. The FBS comparison among the study groups showed a statistically significant difference between groups (p = 0.035).

Groups B (36.8%) and A (28.57%) had the highest blood pressure levels among the groups (p = 0.001), whereas group C had the lowest level of gestational hypertension (0%). The groups did not differ significantly in terms of preeclampsia (p = 0.08); however, the rate of preeclampsia was higher in groups B and A than in the other groups. The groups were similar regarding the other maternal outcomes, including amniotic fluid, abortion, perinatal outcomes, term, and preterm deliveries, delivery type, and pregnancy termination method. One case of stillbirth was observed in group A, and, overall, the rate of cesarean section in all groups was higher than normal vaginal delivery (Table III).

11 cases of multiple pregnancies were reported that did not differ statistically between groups (Table II). Neonatal outcome was evaluted only on single pregnancies. According to the obtained results, there was no significant difference in the neonatal outcomes among the groups, including the 5-min Apgar score, birth weight, neonatal icter, and frequency of anomalies in newborns (Table IV).

**Table 1:** Frequency distribution of PCOS phenotypes in the study participants (n = 198)


**Phenotypes**	**Frequency (n)**	**Percent (%)**
**A**	45	23
**B**	27	14
**C**	52	26
**D**	74	37

**Table 2:** Comparison of demographic characteristics among the study groups


**Variables**		**Group A (n = 45)**	**Group B (n = 27)**	**Group C (n = 52)**	**Group D (n = 74)**	**P-value**
**Age (mean ± SD) (year)**		28.7 ± 5.2	28.7 ± 2.9	28.5 ± 4.9	30 ± 4.7	0.32${}^{*}$
**History of abortion n (%) **		22 (48.9)	11 (40.7)	12 (23.1)	26 (35.9)	0.63${}^{**}$
History of infertility n (%) **		23 (51.1)	6 (22.2)	13 (25)	18 (24.3)	0.007${}^{**}$
**Abortion**		10 (22.22)	8 (29.62)	8 (15.38)	11 (14.86)	0.156**
**Pregnancy method n (%) **						
	**Natural**	34 (75.6)	23 (85.2)	43 (82.7)	62 (83.8)	
	**Medication**	8 (17.8)	4 (14.8)	7 (13.5)	8 (10.8)	
	**IUI**	0 (0)	0 (0)	2 (3.8)	1 (1.4)	
	**ART**	3 (6.7)	0 (0)	0 (0)	3 (4.1)	<brow>-4</erow> 0.44${}^{**}$
**Type of pregnancy n (%)**						
	**Singleton **	31/35 (88.57)	19/19 (100.00)	41/44 (93.2)	59/63 (93.2)	
	**Twin**	3 (8.6)	0 (0.00)	3 (6.8.)	4 (6.3)	
	**Triplet**	1 (2.2)	0 (0.00)	0 (0)	0 (0)	<brow>-3</erow> 0.50${}^{**}$
*ANOVA test, **Chi-squar test						

**Table 3:** Comparison of maternal outcomes in study groups ongoing pregnancies according to phenotype


**Maternal outcomes / Ongoing pregnancy**		**Group A (n = 35)**	**Group B (n = 19)**	**Group C (n = 44)**	**Group D (n = 63)**	**P-value**
**FBS (mean±SD)**		93.65 ± 15.23	89.21 ± 9.80	86.38 ± 12.23	87.31 ± 9.51	0. 035 *
**Abnormal GTT n (%)**		18/35 (51.42)	6/19 (31.57)	13/44 (29.54)	13/63 (20.63)	0.018**
**GDM n (%)**		18/35 (51.42)	6/19 (31.57)	14/44 (31.81)	14/63 (22.22)	0.032**
**Gestational hypertension n (%)**		10/35 (28.57)	7/19 (36.84)	0/44 (0)	9/63 (14.28)	<0.001**
**Preeclampsia n (%)**		6/35 (17.1)	5/19 (26.3)	3/44 (6.8)	5/63 (7.9)	0.08**
**Amniotic fluid n (%)**						0.62**
	**Normal **	32/35 (91.4)	17/19(89.5)	41/44 (93.2)	60/63 (95.23)	
	**Oligohydramnios **	1/35 (2.9)	1/19 (5.3)	3/44 (6.8)	2/63 (3.17)	
	**Polyhydramnios **	2/35 (5.7)	1/19(5.3)	0/44 (0)	1/63 (1.58)	
**PROM n (%)**		3/35 (8.6)	2/19 (10.5)	1/44 (2.3)	4/63 (6.3)	0.54**
**Perinatal outcomes n (%)**						
	**Live births **	34/35 (97.1)	19/19 (100)	44/44 (100)	63/63 (100)	
	**Still birth **	1/35 (2.9)	0/19 (0)	0/44 (0)	0/63 (0)	<brow>-2</erow> 0.30**
**Type of delivery n (%)**						
	**Term **	22/35 (62.85)	12/19 (63.15)	37/44 (84.09)	48/63 (76.19)	
	**Preterm **	13/35 (37.14	7/19 (36.84)	7/44 (15.90)	15/63 (23.80)	<brow>-2</erow> 0.08**
**Pregnancy termination method n (%)**						
	**NVD **	14/35 (40)	7/19 (36.8)	21/44 (47.7)	30/63(47.61)	
	**Cesarean section **	21/35 (60)	12/19 (63.2)	23/44 (52.3)	33/63 (52.38)	<brow>-2</erow> 0.76**
FBS: Fasting blood sugar; GTT: Glucose tolerance test; GDM: Gestational diabetes mellitus; PROM: Premature rupture of membranes; NVD: Normal vaginal delivery. *ANOVA test; **Chi-square test						

**Table 4:** Neonatal outcomes in pregnant women with different PCOS phenotypes


**Variables/Single pregnancy**		**Group A (n = 30)**	**Group B (n = 19)**	**Group C (n = 41)**	**Group D** **(n = 59)**	P-value*
**5-min apgar score (mean±SD)**		9.63 ± 0.88	9.57 ± 1.01	9.63 ± 0.86	9.83 ± 0.56	0.463
**Neonatal birth weight **						
	**AGA **	26/30 (86.66)	16/19 (84.21)	35/41 (85.36)	54/59 (91.52)	
	**Severe IUGR **	2/30 (6.66)	1/19 (5.26)	3/41 (7.31)	0/59 (0)	
	**SGA **	0/30 (0)	1/19(5.26)	1/41 (2.43)	1/59 (1.69)	
	**Macrosomia **	2/30 (6.66)	1/19 (5.26)	2/41 (4.87)	4/59 (6.78)	<brow>-4</erow> 0.733
**Neonatal icter n (%)**		17/30 (56.66)	8/19 (42.10)	14/41 (34.14)	30/59 (50.84)	0.225
**Neonatal condition n (%)**						
	**Good **	25/30 (6.93)	13/19 (68.42)	35/41(85.36)	52/59 (88.13)	
	**NICU admission **	5/30 (16.66)	5/19 (26.31)	6/41 (14.63)	7/59 (11.86)	
	**Neonatal death**	0/30(0)	1/19 (5.26)	0/41 (0)	0/59 (0)	<brow>-3</erow> 0.147
**Anomalies n (%)**		0/30 (0)	1/19 (5.26)	1/41 (2.43)	1/59 (1.69)	0.637
Severe IUGR: a fetal weight below the third percentile for gestational age as determined through an ultrasound. SGA (small gestational age): a fetal weight between the 3${}^{\mathrm{rd}}$ and 10${}^{\mathrm{th}}$ percentiles for gestational age as determined through an ultrasound. *Chi-square test						

## 4. Discussion

In this study, the maternal and neonatal outcomes of different PCOS phenotypes were compared. According to our results, phenotype D (37%) and phenotype B (14%) had the highest and lowest frequencies in the study population, respectively. However, Vaggopoulos and colleagues, in a study on 266 women with PCOS showed that phenotype A was the most frequent (11). Also, Baldani and co-workers obtained similar results in Croatia (12).

In the current research, the abortion rate in phenotypes B and A was higher than in the other phenotypes, but this difference was not statistically significant. According to previous studies, the risk of abortion in women with PCOS is three times higher than in healthy pregnant women (13). Similarly, Palomba and colleagues demonstrated that the abortion rate in phenotype A is significantly higher than in other phenotypes (14). However, Jakubowicz *et al.* did not report any differences among the PCOS phenotypes regarding the abortion rate (15). Perhaps it would be possible to achieve more reliable results on the abortion rate in these patients with a higher sample size of different PCOS phenotypes.

Our results also showed that the rate of GDM in women with PCOS significantly differed among the four phenotypic groups, and the highest GDM level was observed in group A. According to the logistic regression analysis, phenotypes A and B had a higher chance of having a higher mean of fasting blood glucose, OGTT, and GDM compared with phenotypes C and D. In Palomba and co-workers' study, GDM was more frequent in phenotypes A and C (14), whereas,, another in 2015 showed that the incidence of GDM in phenotypes A and B was higher than in the other phenotypes (16).

Furthermore, our study results showed that phenotypes B and A had the highest levels of pregnancy-induced hypertension. A previous study demonstrated that the incidence of maternal and neonatal complications in pregnant women with PCOS was higher than in the control group, especially in the phenotypes with oligo-menorrhea and hyperandrogenism. Also, pregnancy-induced hypertension in phenotypes A and B was reported more often than in other phenotypes (17). Moreover, Kollman and colleagues divided PCOS phenotypes into oligo-ovulatory (A, B) and ovulatory (C, D) groups and reported more pregnancy-induced hypertension in groups A and B compared with the other two groups (16).

In the present study, one intrauterine fatal death was observed in group A, which was not statistically different from the other groups. In normal pregnancies, this rate is predicted by mortality in the uterus. Our results were consistent with other studies (18), which reported that PCOS phenotypes have no effect on stillbirths.

Another study in 2013 reported that maternal complications such as preeclampsia in pregnant women with PCOS are significantly higher than in healthy pregnant women (19). In Palomba's study, the incidence of preeclampsia was highest in phenotypes A and B (20). According to Kollmann and others' results, the incidence of preeclampsia in oligo-ovulatory phenotypes is significantly higher than in others (16). In the current research, the incidence of preeclampsia in phenotypes B and A was highest; but, no significant difference regarding preeclampsia was observed among the studied groups. In the logistic regression test, phenotype B had a higher chance of preeclampsia. There were no differences in the other adverse outcomes of pregnancy such as amniotic fluid disorders, premature rupture of membranes, adverse perinatal outcomes, and preterm deliveries between PCOS phenotypic groups, which was consistent with Kollmann and Palomba's studies (16, 17). Moreover, no significant difference was found in the neonatal outcomes investigated among the different phenotypes of PCOS, including the 5-min Apgar score, birthweight, neonatal anomalies, and NICU admission. similar to Kollmann *et al.* results (16).

## 5. Conclusion

Considering the higher risk of GDM and pregnancy-induced hypertension in PCOS phenotypes A and B, women with these phenotypes require more precise prenatal care.

##  Conflict of Interest

The authors declared that there is no conflict of interest.
